# Assessing Performance of Orthology Detection Strategies Applied to Eukaryotic Genomes

**DOI:** 10.1371/journal.pone.0000383

**Published:** 2007-04-18

**Authors:** Feng Chen, Aaron J. Mackey, Jeroen K. Vermunt, David S. Roos

**Affiliations:** 1 Department of Chemistry, University of Pennsylvania, Philadelphia, Pennsylvania, United States of America; 2 Department of Biology, University of Pennsylvania, Philadelphia, Pennsylvania, United States of America; 3 Genomics Institute, University of Pennsylvania, Philadelphia, Pennsylvania, United States of America; 4 Department of Methodology and Statistics, Tilburg University, The Netherlands; Pasteur Institute, France

## Abstract

Orthology detection is critically important for accurate functional annotation, and has been widely used to facilitate studies on comparative and evolutionary genomics. Although various methods are now available, there has been no comprehensive analysis of performance, due to the lack of a genomic-scale ‘gold standard’ orthology dataset. Even in the absence of such datasets, the comparison of results from alternative methodologies contains useful information, as agreement enhances confidence and disagreement indicates possible errors. Latent Class Analysis (LCA) is a statistical technique that can exploit this information to reasonably infer sensitivities and specificities, and is applied here to evaluate the performance of various orthology detection methods on a eukaryotic dataset. Overall, we observe a trade-off between sensitivity and specificity in orthology detection, with BLAST-based methods characterized by high sensitivity, and tree-based methods by high specificity. Two algorithms exhibit the best overall balance, with both sensitivity and specificity>80%: INPARANOID identifies orthologs across two species while OrthoMCL clusters orthologs from multiple species. Among methods that permit clustering of ortholog groups spanning multiple genomes, the (automated) OrthoMCL algorithm exhibits better within-group consistency with respect to protein function and domain architecture than the (manually curated) KOG database, and the homolog clustering algorithm TribeMCL as well. By way of using LCA, we are also able to comprehensively assess similarities and statistical dependence between various strategies, and evaluate the effects of parameter settings on performance. In summary, we present a comprehensive evaluation of orthology detection on a divergent set of eukaryotic genomes, thus providing insights and guides for method selection, tuning and development for different applications. Many biological questions have been addressed by multiple tests yielding binary (yes/no) outcomes but no clear definition of truth, making LCA an attractive approach for computational biology.

## Introduction

The rapid growth in the availability of genome sequence data, from an ever-increasing range of relatively obscure species, places a premium on the automated identification of orthologs to facilitate functional annotation, and studies on comparative and evolutionary genomics. Homologous proteins share a common ancestry, and may be characterized as either orthologs (which evolve by speciation only) or paralogs (which arise by gene duplication) [1], [2]. Orthologs typically retain similar domain architecture and occupy the same functional niche following speciation, while (functionally redundant) paralogs are likely to diverge with new functions through point mutations and domain recombinations [3], [4].

The concepts of orthology and paralogy are well-established in classical and molecular systematics [1], and have been extended to describe more complicated situations associated with extensive gene duplications commonly observed in eukaryotic species [4]–[6]. In- and out-paralogs are analogous to the phylogenetic concepts in- and out-groups, denoting genes duplicated subsequent or prior to speciation, respectively. Recent duplications yield in-paralogs that may exhibit a many-to-one or many-to-many ortholog relationship with genes in the other species (termed co-orthologs).

**Figure 1:**
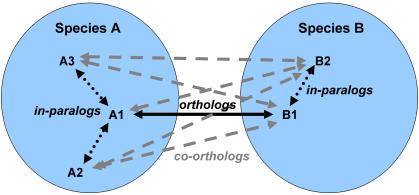
OrthoMCL graph construction between two species, including the establishment of co-ortholog relationships. Solid lines connecting A1 and B1 represent putative ortholog relationships identified by the ‘reciprocal best hit’ (RBH) rule. Dotted lines (e.g. those connecting A1 with A2 and A3, or B1 with B2) represent putative in-paralog relationships within each species, identified using the ‘reciprocal better hit’ rule. Putative co-ortholog relationships, indicated by dashed gray lines, connect in-paralogs across species boundaries (e.g. A3 and B2).

Several strategies have been employed to distinguish probable (co-)orthologs from paralogs, as summarized in Table 1: phylogeny-based methods include RIO (Resampled Inference of Orthology) [7] and Orthostrapper/HOPS (Hierarchical grouping of Orthologous and Paralogous Sequences) [8], [9]; methods based on evolutionary distance metrics include RSD (Reciprocal Smallest Distance) [10], [11]; BLAST-based methods include Reciprocal Best Hit (RBH), COG (Cluster of Orthologous Groups) [12]–[15]/KOG (euKaryotic Orthologous Groups) [15], and Inparanoid [5], [16]. The problem of orthology detection is particularly acute for eukaryotic genomes, because of their large size, the difficulty of defining accurate gene models, the complexity of protein domain architectures, and rampant gene duplications [3], [17]. To address these difficulties, we previously developed the OrthoMCL algorithm [18], which improves on RBH by (*i*) recognizing co-ortholog relationships (Figure 1), (*ii*) using a normalization step to correct for systematic biases when comparing specific pairs of genomes, and (*iii*) using a Markov graph clustering (MCL) algorithm [19] to define ortholog groups. OrthoMCL and Inparanoid exhibit similar performance when comparing two species, but the former is extensible to cluster orthologs across multiple species. Analysis of independently assigned EC number annotations suggests a high degree of reliability [18], and orthology predictions for 55 genomes are available at OrthoMCL-DB [20].

**Table 1:** Various orthology/homology detection methods under study

Methods	Strategy a	Apply to Proteins	Grouping Capability	Parameters Analyzed b	% Positive Protein Pairs	
					Total c	Sampling Average d
**RIO**	Phylogeny	Pfam domains	NO	Orthology bootstrap cutoff	1.9	17.9
**Orthostrapper**	Phylogeny	Pfam domains	NO	Orthology bootstrap cutoff	5.7	39.9
**RSD**	Distance	YES	NO	BLASTP E-value cutoff, Divergence cutoff	2.8	28.8
**RBH**	BLASTP	YES	NO	BLASTP E-value cutoff	5.2	37.7
**Inparanoid**	BLASTP	YES	YES (2 species)	BLASTP E-value cutoff	9.0	43.6
**OrthoMCL**	BLASTP	YES	YES	BLASTP E-value cutoff, MCL inflation index	11.8	52.8
**KOG**	BLASTP	YES	YES	N/A	23.6	66.2
**SBH**	Homology	YES	NO	BLASTP E-value cutoff	11.8	56.6
**BLASTP**	Homology	YES	NO	BLASTP E-value cutoff	41.5	72.1
**TribeMCL**	Homology	YES	YES	BLASTP E-value cutoff, MCL inflation index	47.2	74.7

a Alternative orthology detection strategies (including phylogeny, distance or BLASTP-based), or homology detection methods.

b Parameters analyzed using the LCA benchmarking framework to assess their effect on orthology detection performance (Figure 4).

c The fraction of positively predicted protein pairs (using default parameter settings) within the entire sampling dataset of 567,255 cross-species homologous protein pairs (defined by Pfam domains).

d The average fraction of positively predicted protein pairs (using default parameter settings) from 100 sampling replicates (of the average total of 1590.15 pairs).

Despite the many ortholog identification methods now available, no comprehensive statistical comparison has yet been reported, in part because the lack of a genomic-scale error-free ‘gold standard’ dataset makes it difficult to analyze performance. Functional genomics data are often used as a surrogate for true orthology, both for ortholog assignment (i.e. functional orthologs) [21] and performance assessment [18], and have been used to benchmark a small selection of orthology detection methods, and transfer of functional annotations [22]. Such data are likely to result in many errors, however, especially when applied across large evolutionary distances [4].

Even in the absence of a reliable gold-standard, the comparison of results from alternative methodologies contains useful information, as agreement enhances confidence (provided that the methods employed are independent), and disagreement indicates possible errors (either false positives or false negatives). Latent Class Analysis (LCA) is a statistical technique that can exploit this information, and has been widely applied to multivariate categorical data in research of medical diagnostics, marketing, sociology, etc [23], [24]. For example, when no single, reliable diagnostic test is available for determining the status (latent class) of individuals with respect to a certain disease, LCA can be used to estimate the accuracy (sensitivity and specificity) of multiple diagnostics.

We have applied LCA to the evaluation and optimization of a comprehensive set of orthology detection methods, providing a guide for selecting methods and appropriate parameters. This study also provides an analysis of similarities and statistical dependence between these methodologies. Two widely used ortholog grouping methods – the manually curated KOG database and the automated OrthoMCL algorithm – are further compared with respect to the consistency of clustering, protein function, and protein domain architecture. To illustrate the relationships between orthology and homology detection methods, some other methods BLASTP [25], SBH (Single-way or One-way Best Hit) and TribeMCL [26] were also included in the analysis.

## Results

### Agreement and disagreement between orthology detection methods: input for Latent Class Analysis

A direct comparison of ortholog prediction methods requires a unified dataset, which is difficult to generate due to differences in the data types employed (see Table 1), and differences in the data sources used by published analyses (see Methods). Because KOG groups depend on manual curation, and are therefore not easily updated or recompiled, BLASTP, SBH, RBH, RSD, Inparanoid, OrthoMCL, and TribeMCL analyses were based on the KOG sequence dataset. RIO and Orthostrapper make predictions based on Pfam domains rather than full-length protein sequences; hence proteins lacking Pfam domains were excluded. After mapping Pfam domains to the KOG sequence set, the net result was a dataset containing 27,562 protein sequences from six eukaryotic genomes (*Arabidopsis thaliana, Caenorhabditis elegans, Drosophila melanogaster, Homo sapiens, Saccharomyces cerevisiae*, and *Schizosaccharomyces pombe*), representing 1708 Pfam protein families.

**Figure 2:**
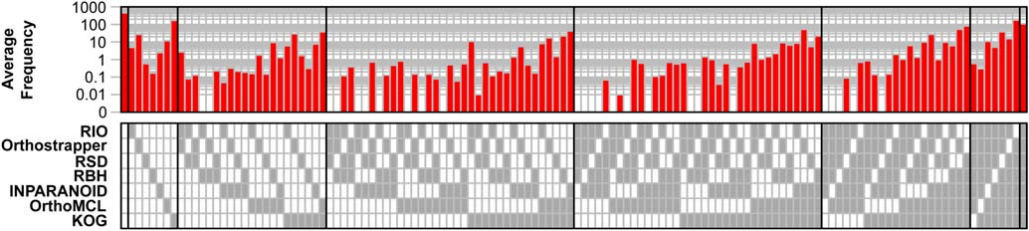
Agreement/disagreement between prediction results of seven orthology detection methods. Average counts of protein pairs identified in 100 sampling replicates are shown (top; note log scale), for each of the 128 (2^7^) possible orthology prediction patterns indicated by filled and empty boxes (bottom), representing positive and negative orthology predictions, respectively.

Cross-species homologous protein pairs (defined as belonging to the same Pfam families) were examined in LCA analysis. Whether or not a given pair of proteins is truly orthologous is unknown (a latent class), but each of the methods under consideration makes a yes/no prediction as to orthology, yielding a pattern comprised of 1's and 0's representing the predictions from all methods. For methods that do not explicitly make predictions for protein pairs, orthology is defined based on clustering into the same groups (for KOG, OrthoMCL, TribeMCL) or sharing of at least one orthologous domain (for Orthostrapper, RIO). The results for a large set of cross-species homologous protein pairs may be summarized as a frequency table (Figure 2). Given such data, the likelihood function for a Latent Class model can be expressed in terms of the overall orthology probability, and the false positive (FP) and false negative (FN) error rates for each method (see Methods). A maximum likelihood estimate of these model parameters is used to represent performance evaluation. In order to avoid biasing the analysis in favor of large protein families, only one cross-species protein pair was sampled from each Pfam family, with 100 replicates of each experiment (see Methods and Discussion).

Figure 2 presents the pattern of agreement and disagreement in orthology calls (shaded boxes), and the average frequency with which each pattern is observed in 100 experimental replicates (specific numbers for one experiment are provided in Figure S1). The most abundant pattern (first column) represents all sampled protein pairs that no method considers to be orthologs. Similarly, many protein pairs are recognized as orthologs by all methods (right-most column). Other common patterns include protein pairs considered to be orthologs by all methods except RIO, reflecting its possibly high FN rate (next-to-last column), and protein pairs considered to be orthologs by KOG only (column 8), reflecting its possibly high FP rate, as discussed below.

### Similarities and statistical dependence between orthology detection methods

In order to assess similarities and dependence between various methods, Jaccard similarity coefficients, mutual information scores, and Pearson correlation coefficients were calculated for all pairwise comparisons between the seven orthology and three homology identification procedures under consideration. Mutual information (Table 2, top) and Pearson coefficients (Table S1, bottom) are commonly used to measure overall/marginal dependence. As indicated in both measurements, many pairs of methods are significantly correlated (top 10 are highlighted by underlining): for example, the best BLAST hit (SBH) rule shows a relatively high degree of correlation with most other BLAST-based methods. Such marginal dependencies are largely due to the overlap of positive predictions, measured by Jaccard coefficient (Table 2, bottom; defined as the fraction of positive protein pairs identified by either method that are recognized by both).

**Table 2:** Overall dependence and similarity between methods

MI^a^	RIO	Ortho-strapper	RSD	RBH	Inpara-noid	Ortho-MCL	KOG	SBH	BLASTP	Tribe-MCL
JCs^b^										
**RIO**		0.07	0.03	0.04	0.05	0.07	0.06	0.05	0.03	0.02
**Orthostrapper**	0.33		0.10	0.13	0.16	0.16	0.13	0.13	0.07	0.05
**RSD**	0.25	0.45		**0.28**	0.20	0.19	0.13	0.20	0.12	0.07
**RBH**	0.28	0.53	0.67		0.24	**0.31**	0.16	**0.30**	0.16	0.11
**Inparanoid**	0.28	0.59	0.57	0.66		**0.35**	0.22	**0.26**	0.17	0.14
**OrthoMCL**	0.29	0.59	0.52	0.70	**0.77**		**0.26**	**0.31**	0.21	0.18
**KOG**	0.26	0.54	0.43	0.55	0.64	**0.75**		**0.25**	0.18	0.17
**SBH**	0.27	0.56	0.51	0.67	0.70	**0.79**	**0.77**		**0.31**	0.20
**BLASTP**	0.23	0.48	0.40	0.52	0.59	**0.70**	**0.78**	**0.79**		**0.34**
**TribeMCL**	0.22	0.46	0.37	0.49	0.57	0.68	**0.77**	**0.72**	**0.91**	

a MI, mutual information. The mutual information between variables *A* and *B* (in this study, *A* and *B* represent two methods' prediction results) is calculated as *MI(A,B) = H(A)+H(B)−H(A,B)* where 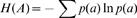 and 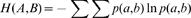 [*p*(*a*) and *p*(*a,b*) are marginal and joint probability distributions, respectively]. The ten highest values are underlined.

b JCs, Jaccard coefficients. The Jaccard coefficient between binary variables *A* and *B* (in this study, *A* and *B* represent two methods' prediction results) is calculated as *JC*(*A,B*) = *p*(1,1)/(1−*p*(0,0)) where *p*(*a,b*) is the joint probability distribution of *A* and *B*. The ten highest values are underlined.

**Figure 3:**
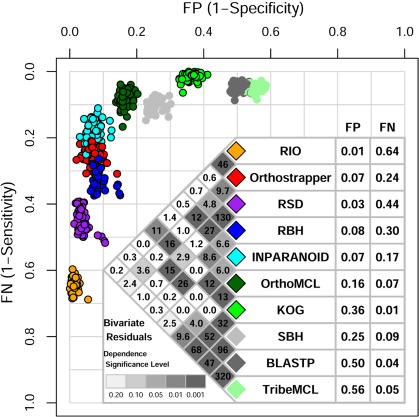
False positive and false negative rates for multiple orthology/homology detection methods. Shaded diamonds present bivariate residual (BVR) statistics calculated based on the orthology data (see Figure 2) and the 2LC model, showing conditional dependence between the ten methods under study. For benchmarking purpose, the CFactor 2LC model is applied to all orthology detection methods to correct for these dependencies (see Figure S2). FP and FN estimates for each method and the overall orthology probability (estimated to be 0.48) are calculated based on the average frequency table from 100 sampling replicates. Those replicates exhibiting a good fit to the CFactor 2LC model (L-square<170) are plotted as colored circles (for illustrative purposes only; FP and FN rates in the table are based on all replicates).

The basic latent class model 2LC (see Methods) assumes independence between methods conditionally on the latent orthology status, commonly referred to as the local independence assumption [24]. This assumption may be violated, however, when two methods make similar errors, i.e. if they yield the same false positive or false negative predictions of orthology. Bivariate residual (BVR) statistics [27], [28] are used to identify possible conditional dependencies among orthology detection methods, as shown in Figure 3. Each BVR corresponds to a Pearson chi-square statistic, comparing observed and expected cross-classification frequency tables for a pair of methods. Although it is only strictly appropriate to compare methods intended to predict orthology, homology detection methods (BLASTP, SBH, TribeMCL) have also been included in the LCA analysis for the sake of illustration.

As indicated in Figure 3 (shaded diamonds), orthology detection methods exhibit various degrees of conditional dependence with each other, particularly when they employ similar strategies. For example, the phylogeny-based methods RIO and Orthostrapper – both of which use the neighbor-joining algorithm for tree construction and calculate confidence based on bootstrapping – show a high level of dependence (BVR = 46.4; *p*<10^−3^). A lower degree of dependence (*p*<0.05) is also observed for most pairwise comparisons between BLAST-based orthology detection methods (RBH, Inparanoid, OrthoMCL, KOG), all of which rely on RBH as their first step. Interestingly, RBH and KOG exhibit extremely low conditional dependence, probably because numerous non-RBH BLAST hits were included during KOG clustering, diluting the dependence signal. RSD and RBH display a high degree of dependence, despite using slightly different measures of sequence similarity, because both are based on a similar rationale of reciprocally identifying the most similar proteins across two genomes. Extremely high dependence is observed between the homology detection methods BLASTP and TribeMCL. The dependence between homology and orthology detection methods is generally very low, except that KOG exhibits much higher dependence with homology detection methods than orthology detection methods, indicating that KOG prediction is very much like a homolog clustering.

Because the conditional dependencies observed between ortholog identification methods could compromise the fit of the basic 2LC model to our orthology data, the CFactor 2LC model (similar to [29], [30]; see Methods) was employed. In this approach, a continuous latent factor with test- and class-specific effects is added to the 2LC model, supposedly to account for any effect which may contribute to the above cross-method conditional dependencies. BVR statistics under this model are provided in Figure S2; when compared with the 2LC model, most of the dependencies between methods disappear. The better fit to our data is also revealed by a significant decrease in L-square statistics [27], [28] (2LC model: 533.7; CFactor 2LC model: 104.8).

Applying the CFactor 2LC model to the average frequency table generated from the 100 sampling replicates described above yields FP and FN estimates for each ortholog prediction method under investigation, as listed in Figure 3. The model is also applied to frequency tables obtained from individual replicates, as illustrated by colored dots in Figure 3. Bold dots (with outlines) provide LCA results for methods that make explicit orthology predictions (RIO, Orthostrapper, RSD, RBH, Inparanoid, OrthoMCL, KOG; see Table 1). Fainter dots (not outlined) represent methods for which LCA is not suitable (SBH, BLASTP, TribeMCL), with error rates calculated as rescaled average posteriors based on estimated model parameters.

### Performance of orthology detection methods

From the data presented in Figure 3, it is clear that most methods trade off sensitivity (1-FN) versus specificity (1-FP). For example, orthology detection methods based on phylogeny (RIO, Orthostrapper) or evolutionary distance (RSD) exhibit low FP error rates (1–7%), but high FN error rates (24–64%). This agrees well with anecdotal experience, where it is often impossible to build a reliable tree – but whenever practical, tree-based methods provide an excellent basis for inferring orthology. Conversely, using homology methods (BLASTP, TribeMCL) to infer orthology results in high FP error rates (50–56%) and low FN error rates (4–5%). This is also in accord with anecdotal experience: BLASTP hits typically include true orthologs but also many false positive results. Between these two extremes, BLAST-based orthology prediction methods exhibit a range of FP and FN rates. Although no single ortholog identification method performs perfectly (both FP and FN = 0), two methods display FP and FN<20%, and these may therefore be considered the best performing algorithms: Inparanoid and OrthoMCL.

As noted above, phylogeny-based methods all exhibit a very low FP error rate, because of the stringent criteria used to predict orthology based on phylogenetic trees. However, they display quite different FN error rates (64% for RIO vs. 24% for Orthostrapper), due to their different specific strategies in ortholog identification. RIO seeks to reconcile a gene tree with a fixed species tree, assigning orthology based on inferred speciation/duplication events [31]. Orthostrapper uses a heuristic orthology assignment algorithm different from the classical tree reconciliation strategy, organizing species into evolutionarily distinct groups instead of a fixed species tree [8], [9]. This simplification appears to greatly improve the sensitivity of phylogeny-based methods, without dramatically affecting specificity.

The reciprocal best BLAST hit strategy (RBH), used as first step for most BLAST-based orthology detection methods, displays a low FP error rate (8%), but its inability to recognize many-to-many or many-to-one co-ortholog relationships results in a high FN error rate (30%), as previously expected [4]. LCA analysis clearly displays improved specificity along the path from BLASTP (FP = 50%) to SBH (25%) to RBH (8%). This improvement comes at a cost of (more modest) reductions in sensitivity, however: FN = 4% for BLASTP, 9% for SBH, and 30% for RBH. The relatively high FP rate observed for SBH might be expected, as one-way best hits are frequently not the nearest neighbor [32].

Several BLAST-based methods have sought to improve upon the trade-offs between sensitivity and specificity. By recognizing co-orthologs, Inparanoid reduces the high FN rate of RBH to 17%. Ortholog clustering across multiple genomes provides a further reduction: to 7% for OrthoMCL, and 1% for KOG. Clustering across multiple genomes inevitably bears a cost in terms of increased FP rates, however: 16% for OrthoMCL, and 36% for KOG. Among all the methods under investigation, KOG displays the best sensitivity (FN = 1%), probably the benefit of extensive manual curation, but at a cost of low specificity (FP = 36%), consistent with the high degree of conditional dependence observed between KOG and homology detection methods (BLAST, TribeMCL).

RSD and RBH are based on a similar concept: reciprocal identification of the most similar proteins between two genomes, and display the highest level of conditional dependence among orthology detection methods (Figure 3). To test whether observed differences in performance (FN = 30%, FP = 8% for RBH; vs FN = 44%, FP = 3% for RSD) might be attributable to the alternative methods used for alignment (local BLAST [25] vs global ClustalW [33]), RBH and RSD analyses are simulated using identical Pfam alignments (see Methods). Pfam_RBH and Pfam_RSD yield virtually identical results (FP = 11% vs. 12%; FN = 36% vs. 39%). Further analysis indicates that the performance of RBH is also affected by the definition of ‘best-hit’: the KOG BLAST analysis used for this study defines E-values<10^−99^ = 0, producing many ties and resulting in a relatively low FN (and high FP) rate. Increasing stringency by using the best similarity score (less ties) increases FN to 38% and reduces FP to 4% (Table S2), close to the values observed for RSD. The divergence threshold parameter used by RSD has no significant effect on performance (Table S3).

### The effect of parameter alteration on orthology detection performance

The perspectives on benchmarking of orthology detection methods provided by LCA suggest that this approach may also be useful for evaluating user-configurable parameters associated with the various methods under study. The following parameters are evaluated: orthology bootstrap cutoff is varied for phylogeny-based methods (RIO, Orthostrapper), BLAST E-value or score cutoff is varied for BLAST-based methods (RBH, Inparanoid, OrthoMCL, etc.), and the MCL inflation index is varied for Markov clustering methods (OrthoMCL, TribeMCL). For each of these methods, only the result generated from default settings (see Methods) is modeled using LCA; for non-default results, error rates are calculated as rescaled average posteriors based on the estimated model parameters.

**Figure 4:**
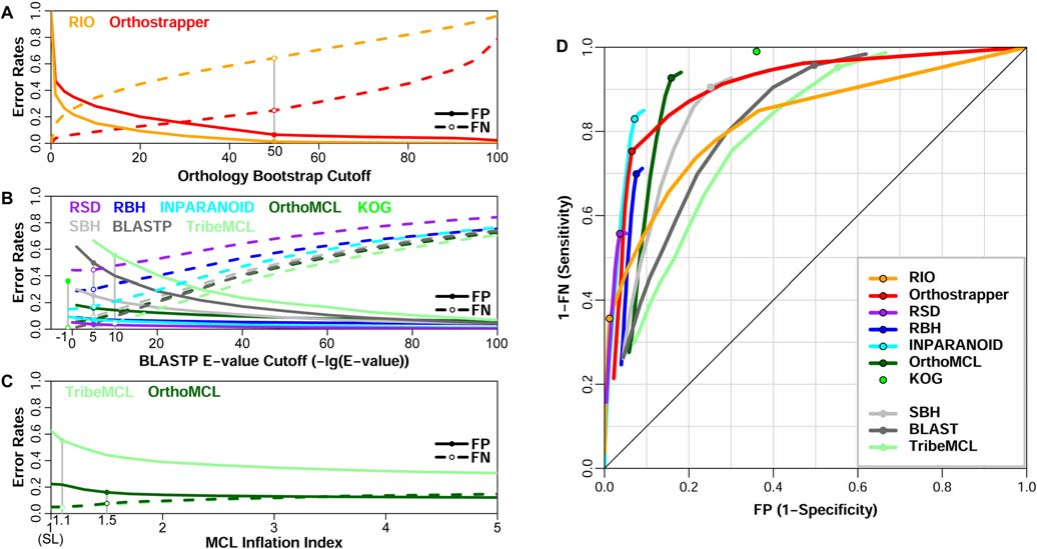
The effects of parameter alteration on orthology detection performance. *Panel A:* Phylogeny-based methods. Varying the orthology bootstrap cutoff indicates that the cross-over point where FP = FN occurs at a lower cutoff than the suggested default (50%; gray bar). *Panel B:* BLAST-based methods. The effect of changing E-value cutoff for various methods (the bit score cutoff used by Inparanoid is transformed into E-value cutoff) is shown. Single data point is provided for KOG, which could not be readily rerun under diverse conditions. *Panel C:* Markov clustering methods. The effect of varying the MCL inflation index is shown. The inflation index of 1 corresponds to single-linkage (SL) clustering. In panels A–C, FP and FN error rates are represented by solid and dashed lines, respectively. *Panel D:* An ROC curve representing the range of FP and FN error rates observed in panels A & B. Default or recommended settings for each method are indicated by circles.

In phylogeny reconstruction, bootstrapping analysis is used to evaluate tree reliability by resampling columns in a multiple sequence alignment. For orthology detection methods, bootstrap values are calculated as the percentage of bootstrapped trees in which two sequences are identified as orthologs, i.e. a confidence measure for orthology prediction. Figure 4A illustrates how the FP and FN error rates of RIO and Orthostrapper vary according to the orthology bootstrap cutoff. This analysis indicates that selecting bootstrap cutoff values lower than the recommended default setting of 50% results in considerable enhancement of sensitivity, with little reduction of specificity (especially for RIO). Orthostrapper differs from RIO primarily in terms of the FN error rate, probably due to the reduction of a multilevel species tree to simpler phylogenetic groupings.

For BLAST-based methods, reducing the E-value cutoff improves specificity and decreases sensitivity (lower FP, higher FN), as shown in Figure 4B. Homology detection methods (BLASTP, TribeMCL) are more sensitive to the E-value cutoff than orthology detection methods in FP rate – especially at high E-values, where ortholog protein pairs are rare. For example, changing the E-value cutoff from 0.1 to 0 reduces FP rates by 57% for BLASTP, but only 5% for RBH.

As graph-based clustering algorithms, both OrthoMCL and TribeMCL apply Markov clustering to identify groups from an all-against-all protein similarity graph. Accurate identification of clusters should eliminate incorrect edges, such as those introduced due to protein domain fusion or other rearrangements. Increasing the inflation index parameter increases cluster tightness, thus reducing FP while raising FN, as shown in Figure 4C. The MCL inflation index has only a modest effect on performance of OrthoMCL, as previously described [18]. For example, increasing the inflation index from 0 (single-linkage clustering) to 5 reduces FP by 10% for OrthoMCL but 32% for TribeMCL. The greater impact on TribeMCL is attributable to its denser protein connectivity graph, incorporating edges between all homologs defined by BLAST hits, while the OrthoMCL graph only contains edges representing (co-)ortholog and in-paralog relationships (Figure 1).

The effect of altering orthology bootstrap and BLAST similarity cut-offs is best illustrated by an ROC (Receiver Operating Characteristic) curve, as shown in Figure 4D. The sensitivity/specificity trade-off is readily seen in this figure, although the entire range cannot be explored for all methods using standard parameters. It is interesting to note that for phylogeny-based methods, the recommended parameter settings (indicated by circles) sacrifice sensitivity in favor of high specificity, a consequence of arbitrarily imposing a relatively high bootstrap cutoff of 50%. In contrast, the KOG method sacrifices specificity for sensitivity (only a single value is shown for KOG, as this manually curated method is not readily repeated using different parameter settings).

### Comparison of ortholog/homolog groupings

Only three of the methods under investigation (KOG, OrthoMCL, TribeMCL) permit clustering proteins from multiple species, rather than simply making pairwise predictions for proteins from two species. As shown in Table 3, from the entire dataset of 112,920 sequences, KOG clusters 88,613 proteins (78%) into 10,058 groups (average 8.8 proteins/group); TribeMCL clusters 83,219 proteins (74%) into 8143 groups (average size 10.2); and OrthoMCL clusters 78,998 proteins (70%) into 13,323 groups (average size 5.9). Thus, in terms of inclusiveness, KOG>TribeMCL>OrthoMCL, while in terms of cluster tightness, OrthoMCL>KOG>TribeMCL. Not surprisingly, there is considerable overlap in which proteins are grouped by these methods; 76,114 (67%) proteins are grouped by all three methods. When comparing the two methods that explicitly define ortholog groups (KOG & OrthoMCL), the vast majority of the extra 3265 (13,323–10,058) OrthoMCL groups contain≤4 sequences, from 1–2 species (see Figure S4 for a distribution of group sizes using each method, by # species and # sequences).

**Table 3:** Comparison of proteome clusterings by OrthoMCL, KOG, and TribeMCL^a^

	A	B	Grouped By Both	Identical Groups	A Contains B	B Contains A	Coherent Groups^b^
	**OrthoMCL**	**KOG**					
**# Groups**	13,323	10,058		5,158	474/572	7,059/3,597	12,691/9,327
				(39%/51%)^d^	(4%/6%)^d^	(53%/36%)^d^	(95%/93%)^d^
**# Proteins**	78,998	88,613	78,329	22,057	7,106/3,327	42,398/58,997	71,561/84,381
	(70%)^c^	(78%)^c^	(99%/88%)^d^	(28%)^e^	(9%/4%)^d^	(54%/67%)^d^	(91%/95%)^d^
	**OrthoMCL**	**TribeMCL**					
**# Groups**	13,323	8,143		5,116	859/977	6,421/1,895	12,396/7,988
				(38%/63%)^d^	(6%/12%)^d^	(48%/23%)^d^	(93%/98%)^d^
**# Proteins**	78,998	83,219	76,625	20,290	6,310/4,722	45,002/56,739	71,602/81,751
	(70%)^c^	(74%)^c^	(97%/92%)^d^	(26%)^e^	(8%/6%)^d^	(57%/68%)^d^	(91%/98%)^d^
	**KOG**	**TribeMCL**					
**# Groups**	10,058	8,143		4,289	1,914/2,711	2,398/854	8,601/7,854
				(43%/53%)^d^	(19%/33%)^d^	(24%/10%)^d^	(86%/96%)^d^
**# Proteins**	88,613	83,219	81,860	21,140	31,040/15,842	21,039/40,954	73,219/77,936
	(78%)^c^	(74%)^c^	(92%/98%)^d^	(26%)^e^	(35%/19%)^d^	(24%/49%)^d^	(83%/94%)^d^

a Total proteome size = 112,920, of which 76,114 (67.4%) were grouped by all three methods.

b Coherent groups includes cases where methods A and B yield identical groups, cases where a group identified by one method completely encompasses one or more groups identified by the other method (i.e. the sum of the preceding three columns).

c Percent of total proteome.

d Percent of those groups or proteins that were clustered by method A (left) or B (right).

e Percent of those proteins that were clustered by both methods.

Table 3 also presents further analysis of the relationship and coherence of KOG, OrthoMCL, and TribeMCL clusterings. Comparing the two ortholog groupings (KOG & OrthoMCL; top row), of the 78,329 sequences grouped by both methods, 90–95% are grouped coherently (right-most column), i.e. KOG and OrthoMCL groups are either identical or one is completely contained within the other. 5158 identical groups represent 51% of KOG groups and 38% of OrthoMCL groups, and include 28% of all proteins recognized by both. 35% of KOG groups contain 52% of the OrthoMCL groups, while only 3% of OrthoMCL groups contain 5% of KOG groups, i.e. KOG groups generally encompass OrthoMCL groups, rather than the reverse (note that in a previous analysis, OrthoMCL groups were found to generally encompass EGO groups [18], [34]). A similar trend is also observed when comparing OrthoMCL & TribeMCL, but not KOG & TribeMCL, as shown in Table 3. Only 84 OrthoMCL groups are split into two (or more) groups by KOG; these are often attributable to functionally distinct proteins that are not distinguishable by sequence similarity (e.g. RNA polymerases I and III), or fusion proteins (e.g. in the case of bifunctional ATP sulfurylases - adenosine 5′-phosphosulfate kinases, these related enzymes were manually split into individual monofunctional groups and a separate bifunctional group during curation of the KOG database).

### Consistency of protein function and domain architecture in ortholog/homolog groupings

As described above, OrthoMCL and KOG are very consistent in their grouping of proteins from a multi-species dataset, although the former is fully automated, while the latter requires manual curation. In general, differences are attributable to the tendency of KOG to incorporate into larger groups proteins that are either excluded or grouped separately by OrthoMCL – but which of these clusterings is more accurate? By definition, orthologs arise through speciation, and are likely to retain similar sequence, domain architecture, and function; indeed, such conservation provides one motivation for identifying ortholog groups, in order to facilitate the annotation of unknown protein sequences. In contrast, paralogs arise by duplication; because of their functional redundancy, they are more likely to have point mutation and even domain rearrangements to evolve new functions. The consistency of protein function and domain architecture within ortholog/homolog groups therefore provides a useful measure for assessing the accuracy of ortholog groupings.

Enzyme commission (EC) numbers are among the most widely and consistently applied forms of curated functional annotation for proteins. In order to investigate the accuracy with which ortholog identification algorithms cluster EC-annotated proteins, protein sequences and EC numbers were extracted from the ENZYME database, and mapped to the dataset used for ortholog analysis (see Methods). Complete (4-digit) EC numbers are identified for a total of 4,739 sequences in the dataset, >95% of which are clustered into groups by OrthoMCL, KOG and TribeMCL. Groups containing two or more EC-annotated sequences (defined as enzyme groups) are used to assess functional consistency, by examining the percentage of enzyme groups for which all EC annotations are identical or consistent.

As shown in Table 4, OrthoMCL recognizes more enzyme groups, containing fewer proteins, than either KOG or TribeMCL, due to the different tightness of these clusterings. As a consequence, OrthoMCL exhibits the highest consistency in EC number annotation: 89% of enzyme groups, vs. 83% for KOG, and 75% for TribeMCL. Of 4125 EC-annotated sequences in OrthoMCL enzyme groups, 3531 (86%) are clustered into consistent groups (vs. 77% for KOG and 54% for TribeMCL). From another point of view, these statistics indicate that OrthoMCL exhibits the greatest potential for accurate functional annotation of unknown protein sequences.

**Table 4:** Consistency of three clustering methods with EC assignments

Method	Total Dataset		Enzyme Groups^b^			Consistent Enzyme Groups^d^		
	Groups	Proteins (% of proteomes)^a^	Groups	Proteins	EC-annotated (% of total)^c^	Groups (% possible)	Proteins	EC-annotated (% possible)
**OrthoMCL**	13,323	78,998 (70)	1,007	10,371	4,125 (87)	895 (89)	8,081	3,531 (86)
**KOG**	10,058	88,613 (78)	926	14,471	4,393 (93)	773 (83)	9,963	3,378 (77)
**TribeMCL**	8,143	83,219 (74)	639	19,685	4,437 (94)	481 (75)	7,387	2,388 (54)

a Total proteome size = 112,920.

b Enzyme groups are defined as groups with at least two proteins for which EC annotation is available.

c A total of 4,739 proteins have EC annotations according to ENZYME database.

d All EC-annotated proteins in the group have the same or consistent EC numbers. Percentages indicate fraction of enzyme groups which are consistent in EC annotation, or fraction of EC-annotated proteins properly put into consistent groups.

Several strategies can be employed to define protein domain architecture, often motivated by the desire to identify specific domains that are structurally (SCOP, CATH) and/or functionally (Pfam) conserved. In order to incorporate regions that may not be structurally or functionally significant, we applied MKDOM2 [35], which decomposes all protein sequences into domains based on successive iterations of PSI-BLAST searches. After excluding domains that are present in only one sequence in the entire dataset, a ‘Domain Content Similarity’ (DCS) Jaccard coefficient is defined as the number of domains present in both of two sequences, divided by the number of domains present in either (for a group of sequences, DCS refers to the average of all pairwise comparisons).

**Figure 5:**
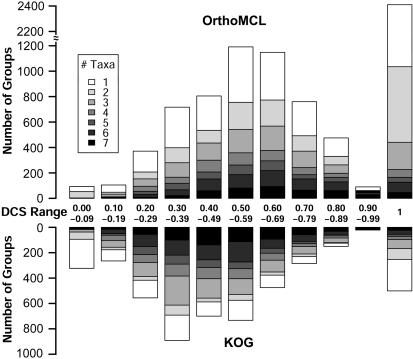
Comparison of protein domain content similarity for OrthoMCL and KOG groups. The distribution of Domain Content Similarity (DCS) values for non-identical KOG and OrthoMCL groups is shown. Shading is used to represent group size (number of taxa). In general, OrthoMCL groups are smaller, and exhibit more consistency in protein domain architecture.

A large amount of groups (33∼47%) identified by OrthoMCL, KOG or TribeMCL exhibit DCS = 1 (see Figure S5), i.e. they share all of the same domains (ignoring domain order, length, or repetition). Considering those groups that are not identical between OrthoMCL and KOG, the average DCS is significantly higher for OrthoMCL, as shown in Figure 5: 4-fold more OrthoMCL groups exhibit DCS = 1, and for those groups with non-identical domain architectures, the peak is shifted to the right from KOG (DCS = 0.3–0.4) to OrthoMCL (0.5–0.6). This partly explains OrthoMCL's better consistency in protein function, as higher similarity in protein domain architecture results in higher similarity in function [36]. As might be expected from the overall comparison of OrthoMCL vs KOG consistency (Table 3), the more consistent OrthoMCL groups are also smaller in size, as indicated by shading in Figure 5. Smaller groups with more consistent DCS are less likely to include out-paralog evolutionary relationships.

**Figure 6:**
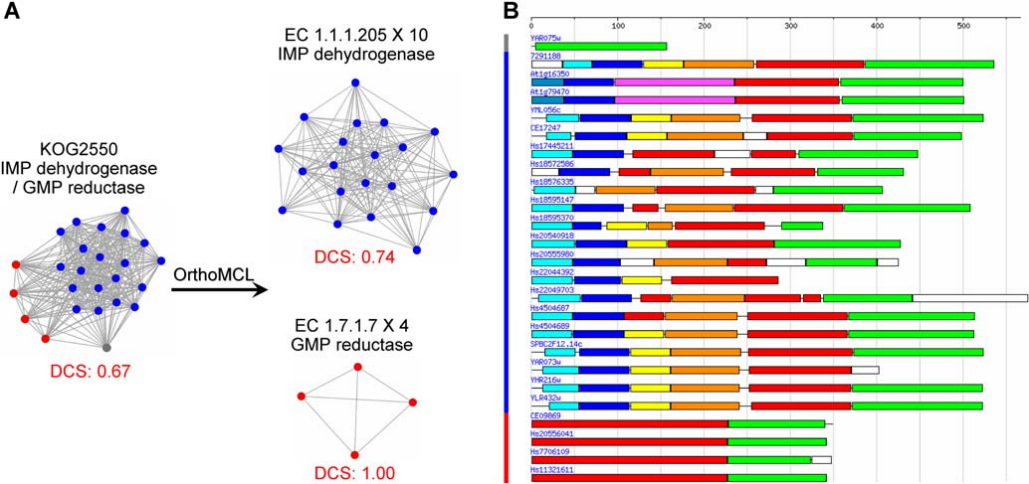
Example of KOG vs OrthoMCL clustering. Group KOG2550 is split by OrthoMCL into two groups that are more consistent with respect to both EC annotation and protein domain architecture. *Panel A:* Edge lengths in these two BioLayout graphs [40] indicate BLAST similarity relationships, and node colors represent different OrthoMCL groups (note that one protein, shown in gray, is not clustered by OrthoMCL). OrthoMCL uses normalized BLAST scores [18], and clustering is based on the identification of (co-)orthologs and in-paralogs (Figure 1), rather than simply homologs defined by BLAST. *Panel B:* Colored vertical bars correspond to OrthoMCL groups; colored horizontal bars indicate conserved domains assigned by MKDOM2 [35].

In summary, OrthoMCL yields more, smaller protein groups than other methods, and is more effective in separating ancestral duplications (out-paralogs). These groups are more consistent with respect to both EC number annotation and protein domain architecture, and are therefore more likely to accurately reflect protein evolution and function. For example, group KOG1158 includes some proteins annotated with EC 1.1.1.205 (IMP dehydrogenase), and others annotated as EC 1.7.1.7 (GMP reductase). OrthoMCL successfully clusters these proteins into two groups with consistent EC numbers (Figure 6A) and domain architectures (Figure 6B). Both enzymes share two protein domains, but IMP dehydrogenase also includes a long N-terminal extension, incorporating several additional domains.

## Discussion

### Performance of orthology detection methods

Ortholog identification is critically important for many applications, ranging from genome annotation to comparative genomics to evolutionary biology. We have therefore sought to develop a platform for comparing available ortholog identification methods. In the absence of a genomic-scale orthology dataset suitable for benchmarking performance, the statistical technique of Latent Class Analysis allows false positive and false negative rates to be inferred from data on agreement and disagreement (Figure 2). Applying this approach to a variety of ortholog identification methods (Table 1) demonstrates a clear trade-off between sensitivity and specificity, both with respect to the methods themselves (Figure 3) and the parameters selected (Figure 4).

As described above (and in accord with anecdotal experience), phylogeny-based methods typically exhibit high false negative rates, while simple BLAST-based homology detection methods exhibit high false positive rates. A similar trade-off between specificity and sensitivity has recently been described based on the use of functional genomics data to benchmark human-mouse orthology predictions [22]. Of the ten methods studied in our analysis, only four (RBH, Inparanoid, OrthoMCL and KOG) were included in that study: rankings in sensitivity (approximated indirectly as raw numbers of ortholog predictions) and specificity (inferred indirectly based on functional similarity score) partly agree with the present report. However, it should be noted that different types of functional genomics data yield different results, and that such evidence is often inadequate to the challenge of making comparisons over larger evolutionary distances, e.g. *H. sapiens* to *C. elegans* [22].

Table 1 and Figures 3–4 provide a helpful framework for selecting suitable methods for various applications. For example, KOG provides a low false negative rate (but high frequency of false positives), while RIO offers the reverse. KOG is therefore suitable for applications requiring high sensitivity, such as the identification of all candidate genes that might encode a specific enzyme, while RIO is more appropriate for applications requiring high specificity, such as the identification of groups suitable for phylogenetic analysis, or for comparative biochemical studies of enzyme function. Overall, Inparanoid and OrthoMCL exhibit the best balance of sensitivity and specificity.

Other factors may also affect the selection of ortholog identification strategies. For example, RIO and Orthostrapper are based on analysis of aligned Pfam domains. These methods calculate evolutionary distances and reconstruct phylogenies, incurring a relatively high computational cost. All of the other methods considered here are based on BLAST comparison of full-length protein sequences, and are therefore relatively fast. The KOG method, however, relies on manual curation to break apart inappropriately combined groups – a labor-intensive task that precludes automated incorporation of emerging genome sequences.

These methods also differ in their ability to group protein sequences from multiple species – a particularly important consideration for such applications as functional genome annotation and phyletic pattern analysis. KOG, OrthoMCL and TribeMCL assemble protein groups from multiple species – the former by merging ‘triangles’ of reciprocal best hits based on shared edges (followed by a variety of heuristic steps designed to improve sensitivity), while the latter two use a Markov clustering algorithm to form groups from a complex graph defined by pairwise sequence similarity scores. Other methods are designed for two-species datasets, although a recent report (MultiParanoid [37]) employs a single linkage clustering on Inparanoid results from all possible bi-species comparisons to group proteins across multi-species dataset (in order to prevent the inclusion of out-paralogs, MultiParanoid is only employed for closely related species). Similar strategies could be applied to other methods as well, although the false positives inevitably brought about by single-linkage clustering make it hard to apply to large number of species.

Groupings formed by three multi-species clustering algorithms are compared in Table 3. In general, KOG and TribeMCL are more inclusive than OrthoMCL, leading to lower group consistency in terms of both protein function (Table 4 & Figure 6A) and domain architecture (Figures 5 & 6B). The inclusive nature of TribeMCL is attributable to its use of sequence similarity scores alone to assign groupings (i.e., this method is intended to identify homologs, in contrast to KOG and OrthoMCL, which purport to identify orthologs only). KOG's inclusive nature is due to a variety of factors, including the requirement for three species (forming a triangle of reciprocal best hits) during the construction of initial seed groups, the use of a high BLAST E-value cutoff (10 for KOG, vs 10^−5^ for OrthoMCL), and permissive rules for adding individual sequences to the initial seeds. By way of example (Figure 6), GMP reductases cannot nucleate a KOG seed group because they are only found in two species in the KOG dataset (*H. sapiens* and *C. elegans*). They are therefore inappropriately grouped with IMP dehydrogenases in KOG2550 due to sequence similarity, but properly separated by OrthoMCL.

### Challenges in using LCA for benchmarking orthology detection

As noted above, LCA provides a useful framework for benchmarking ortholog assignment methods, but such application is not trivial and we have encountered several challenges. LCA compares various methods with each other, allowing error rates to be inferred in the absence of a gold standard. Because error rates estimated in this way may be affected by other methods included in the analysis, we considered which (and how many) methods should be included, the (in)dependence between these methods, and the robustness of the final result obtained.

In order to provide the most comprehensive and accurate analysis possible, seven orthology detection methods are included, representing complementary strategies based on phylogenetic reconstruction, evolutionary distances, or BLAST-based sequence similarity. Jackknife analysis of these results shows little systematic change when any one or two methods are removed from the analysis (Figure S3). The most significant change observed is an apparent improvement in the performance of RBH and RSD when any highly-specific, relatively independent method (RIO, Orthostrapper, Inparanoid, OrthoMCL) is excluded (Figure S3B). Exclusion of individual methods also results in a slight increase in both FP and FN rate estimation for Orthostrapper and RIO, suggesting that the performance of these methods may be slightly overestimated. Overall, while the specific false positive and false negative values estimated by LCA depends on the particular collection of methods examined, this analysis confirms the value of incorporating a wide range of methods in the benchmarking framework, the overall robustness of this analysis, and the utility of LCA as a method for evaluating performance.

Orthology detection methods all tend to rely on a similar set of concepts for identifying protein pairs across species boundaries (Table 1), making cross-method conditional dependence a potential complication (Figure 3). Such local dependence is a common problem confronting the use of LCA in many fields, but methods have been developed to modify basic latent class model by adding an extra latent variable [29], [30]. The application of such model accurately models conditional dependencies between orthology detection methods (Figure S2), and results in a better fit to our orthology data (i.e. improvement in performance estimation).

One significant feature of this LCA application is the consideration of relationships between subjects (“Are two proteins orthologs of each other?”), rather than individual subjects themselves (e.g. “Does a patient have a specific disease?”). Instead of considering all possible pairwise relationships between all proteins in the entire dataset, the frequency table used for LCA input includes only cross-species homologous protein pairs, as orthology can only occur between homologs from different species. Recognizing that orthology detection performance may vary from one protein family to another, a sampling strategy (with replicates) was devised to weight all families equally (see Methods), to prevent the skewing of relationship data in favor of large protein families. Sensitivity and specificity results estimated in this report therefore represent an average over all families.

### Applications of LCA in Computational Biology

LCA methodology is well suited to many biomedical problems, where the inability to define a gold standard or unequivocally recognize truth is a common limitation. This report describes one of the first applications of LCA to computational biology, but the emergence of genomic-scale datasets suggests many other potential applications. For example, numerous computational methods have been devised to predict potential protein-protein interactions, but high-throughput experimental methods typically exhibit a high false positive rate, precluding the development of a well-validated dataset.

In addition to its utility for evaluating test performance, LCA may also be employed as a clustering algorithm, based on the posterior probability of subject membership in each latent class. For example, LCA has been widely used to classify disease status or subtypes, based on various types of symptoms or diagnostic tests. In computational biology, we have exploited LCA as a gene model combiner, integrating diverse lines of evidence to significantly improve eukaryotic gene model predictions [38]. Since available orthology detection strategies display a trade-off of sensitivity and specificity – without any method achieving optimal performance in both – it should be possible to employ a similar clustering strategy for merging ortholog predictions from multiple methods, improving on the performance of any individual one. Although this strategy would undoubtedly be impractical for general application (due to intensive computing requirements), it might be quite useful to generate a close-to-gold-standard genomic-scale orthology dataset, establishing a benchmark for future analyses, and guiding computational and biochemical investigation of ortholog structural and functional properties.

## Materials and Methods

### Orthology detection

The KOG database represents a manually curated grouping of orthologs based on 112,920 protein/domain sequences from seven eukaryotic genomes: *Caenorhabditis elegans, Drosophila melanogaster, Homo sapiens, Arabidopsis thaliana, Saccharomyces cerevisiae, Schizosaccharomyces pombe* and *Encephalitozoon cuniculi*. The protein sequence dataset used in KOG construction was compiled as of July 1, 2002 [39]. In order to facilitate the comparison and evaluation of multiple ortholog identification methods, this dataset was employed for all analyses (except as otherwise noted). Sources for all ortholog identification algorithms are as follows:

#### KOG:

Ortholog grouping data and protein sequence data for the seven eukaryotic genomes noted above were downloaded from the KOG database (http://www.ncbi.nlm.nih.gov/COG/new/).

#### BLASTP:

The BLAST result file was also downloaded from the KOG database. *Default settings for LCA:* E-value cutoff = 10^−5^.

#### OrthoMCL:

Program v1.4 was downloaded from http://orthomcl.cbil.upenn.edu (MCL v02-063 was downloaded from http://micans.org/mcl/) and applied to the KOG dataset using the above KOG BLAST result file. *Default settings for LCA:* BLAST E-value cutoff = 10^−5^; MCL inflation index = 1.5.

#### SBH, RBH:

RBH results were obtained from the OrthoMCL output; SBH results were obtained by modifying the OrthoMCL script. ‘Best-hit’ is defined as the hit (or multiple hits tied) with the highest E-value. *Default settings for LCA:* BLAST E-value cutoff 10^−5^.

#### Inparanoid:

Program v1.35 was downloaded from http://inparanoid.cgb.ki.se and applied to all pairwise species proteomes extracted from the KOG dataset (without the use of outgroup species). *Default settings for LCA:* BLAST bit score cutoff = 50 bits.

#### TribeMCL:

Program v1.6 and MCL v02-063 were downloaded from http://micans.org/mcl/. The KOG BLAST result file was used for TribeMCL clustering. *Default settings for LCA:* BLAST E-value cutoff = 10^−10^; MCL inflation index = 1.1.

#### RSD:

Program was downloaded from http://rodeo.med.harvard.edu/tools/roundup/and applied to all pairwise species proteomes extracted from the KOG dataset (using the KOG BLAST result file). *Default settings for LCA:* BLAST E-value cutoff = 10^−5^; divergence cutoff = 0.8.

#### Orthostrapper:

Predictions on domain sequences of Pfam 7.2 were downloaded from the HOPS database at ftp://ftp.cgb.ki.se/pub/data/HOPS/. *Default settings for LCA:* Orthology bootstrap cutoff = 50.

#### RIO:

Program v1.1 was downloaded from http://www.rio.wustl.edu and applied to domain sequences in Pfam 14.0. Pre-calculated data on evolutionary distance between domain sequences was kindly provided by the authors. *Default settings for LCA:* Orthology bootstrap cutoff = 50.

#### Pfam_RSD:

The RSD strategy was applied to Pfam 14.0, using pairwise distances extracted from the RIO dataset. Reciprocal smallest distance pairs of domain sequences were identified (without using divergence cutoff) for each Pfam domain and each pairwise species comparison.

#### Pfam_RBH:

The RBH strategy was applied to Pfam 14.0. For each cross-species pair of sequences belonging to the same domain, the original Pfam alignment was trimmed to the first and last conserved columns, and the resulting alignment was used to calculate a similarity score (Scoring matrix: BLOSUM62; Gap Open Penalty: 11; Gap Extension Penalty: 1). Reciprocal best hit pairs of domain sequences (for each Pfam domain and each pairwise species comparison) were identified based on similarity scores.

### Frequency tables

Large protein families may bias the assessment of orthology detection performance, by drastically amplifying the number of cross-species homologous protein pairs relative to small protein families. In order to avoid this problem, a sampling strategy was therefore devised to consider only one protein pair, from one species pair, from each Pfam family, chosen at random except for the exclusion of *S. cerevisiae* and *S. pombe* pairs (which are not distinguishable in the Orthostrapper/HOPS predictions [9]), and pairs including *E. cuniculi* (absent from the Orthostrapper/HOPS dataset [9]). As a further restriction, no protein pair was selected more than once in any given sampling (even if present in more than one Pfam families).

### Latent class models and analysis

In orthology detection, both the prediction results *X_i_* (*i = 1,…,n*, representing different methods) and the true status of orthology *Y* (latent class) are binary: 1 for orthology, 0 for non-orthology. For a given homologous protein pair, the result of all these methods is represented as a pattern *X* (*X_1_ X_2_ … X_n_*). The probability of observing a specific pattern *x* can be expressed using *2n+1* parameters: the prevalence rate of orthology *θ* among homologous protein pairs, the FP rate *α_i_* and the FN rate *β_i_* for each method, according to the following formula.1
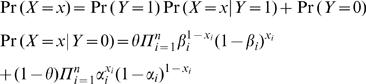



For a set of homologous protein pairs, a frequency table can be compiled, listing the counts of pairs for all the 2^n^ possible patterns as *f(X)*. The likelihood function of the latent class model given this data can be expressed as2
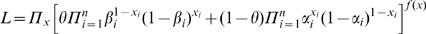



Finally the ML (Maximum Likelihood) estimate of these parameters can be obtained by using Latent GOLD software [27]. This model is a basic 2LC model (i.e. containing 2 latent classes), assuming conditional independence. Once the model parameters are estimated, the probability of observing each prediction pattern can be calculated according to formula 1, above.

In order to account for the conditional dependencies observed between orthology detection methods, an extra latent variable is added to the above basic model [29], [30]. In such models, the outcome of a test is assumed to be governed by two factors: the latent class of orthology status (true or false), and a second latent variable which summarizes the attributes of the subject (here, the homologous protein pair) and the test (here, the orthology detection method) that are not explained by the latent class of orthology status alone. Generally, the second latent variable is in the form of standard normal distribution, and a probit model is used in describing the conditional probability. Thus, such models are also called latent class models with random effects or a continuous factor (a CFactor 2LC model). Given both of these latent variables, the responses of different tests then are assumed to be independent, so the formulas describing this model are similar to those used for the basic model. For a detailed description please refer to [29] or Latent GOLD technical guide [28].

Latent GOLD software version 4.0 was used to perform all LCA analysis in this paper. For the 2LC model, Latent GOLD uses a frequency table file as input, and performs ML estimation of model parameters, by: *(i)* selecting “Cluster Model” with 2 clusters (i.e. two latent classes representing “orthology” and “non-orthology” respectively), *(ii)* setting orthology detection methods as “Indicators”, *(iii)* setting the counts of protein pairs for various prediction patterns in frequency tables as “Case Weight”, and *(iv)* setting 100 random sets as “Start values”, and 250 iterations per set, in ML estimation. The CFactor 2LC model is run similarly, except that a continuous factor (CFactor) is added with “Cluster Dependent” (i.e. latent class dependent) and “Unequal” (i.e. test dependent) effects. The output of this analysis includes estimated values for model parameters, as well as BVR statistics.

BVR statistics calculated under the 2LC model may be regarded as a measurement of conditional dependence. For the purpose of illustrating the relationship between orthology and homology detection methods, both kinds of methods were considered. For evaluating orthology performance, only the seven orthology detection methods (using default parameter settings) were included under the CFactor 2LC model, composing the benchmarking framework. Homology detection methods do not purport to detect orthology, and are therefore inappropriate for inclusion. These methods, as well as orthology detection methods under non-default parameter settings, can be set in LatentGOLD software as “Inactive Covariates” (i.e. they do not play a role in LCA analysis), and their error rates are obtained by rescaling average posteriors to sum to 1 within classes.

### Consistency analysis in protein function and domain architecture

EC annotated sequences were obtained from SWISSPROT ENZYME database at http://us.expasy.org/enzyme/, and mapped to KOG sequence dataset based on exact matches. Only ortholog groups containing at least two sequences for which EC annotation is available were examined, and groups were considered to be consistent if all EC annotations are identical or consistent (i.e. where one enzyme's EC number(s) are contained within the set of EC numbers used to describe the other multifunctional enzyme).

MKDOM2 program was downloaded from http://prodom.prabi.fr/prodom/xdom/and run against the complete KOG sequence dataset using default settings.

## Supporting Information

Figure S1 Frequency table from one sampling replicate. Last column lists the number of protein pairs observed for each orthology prediction pattern. Note that 50% (64) of the 2ˆ7 = 128 possible patterns are not observed in this replicate (the rows are not shown).
(4.01 MB TIF)

Figure S2 Bivariate residual statistics (BVR) calculated based on the CFactor 2LC latent class model. Note that most conditional dependencies decrease significantly in comparison with BVR statistics based on the 2LC model (Figure 3), indicating that they are effectively modeled by the extra latent variable added in the CFactor 2LC model.
(0.42 MB TIF)

Figure S3 Jackknife analysis of orthology detection performance based on LCA. Panel A: Removal of any one or two orthology detection methods from the LCA benchmarking framework (medium- and small-sized circles, respectively) has relatively little impact on performance. Large circles indicate performance assessment when all methods are included (see Figure 3). Note that the overall trend of orthology detection performance is maintained in jackknife analysis. Panel B: Data extracted from panel A to indicate specific effects of (one-) method removal. Data points indicate changes in the estimation of FP & FN error rates for each method (outer circle), with respect to the original benchmarking result, following removal of other methods (inner circle). Most changes observed on these error rates are ≤0.1 (indicating the relative robustness in estimation), but systematic changes of some methods indicate possible errors: RBH's lower FN rates when some methods are removed suggest possible underestimation of sensitivity; phylogeny-based methods RIO and Orthostrapper are concentrated in the upper right quadrant, suggesting possible overestimation of performance.
(2.72 MB TIF)

Figure S4 Size distribution of KOG, OrthoMCL and TribeMCL groups (with respect to # sequences and # species). When compared with KOG and TribeMCL, the majority of extra groups identified by OrthoMCL contain≤4 sequences, from 1–2 species.
(2.05 MB TIF)

Figure S5 Domain structure consistency of OrthoMCL, KOG and TribeMCL groups. DCS (Domain Content Similarity) is defined as a Jaccard coefficient: the fraction of MKDOM2 domains found in either of two sequences that are present in both; DCS for a group of sequences is calculated as the average of all pairwise comparisons of sequences. (Note that the effects of domain repeat, order and length are not considered under this simple definition. In addition, due to the difficulties of accurately predicting gene models, e.g. start codon positions and intron/exon structures, orphan domains appearing only once in the entire dataset are excluded.) Groups from all three methods display a similar distribution in DCS value, with OrthoMCL exhibiting better consistency than KOG (average DCS is 0.73 for OrthoMCL vs 0.65 for KOG). The stringent BLAST E-value cutoff (10ˆ-10) used in TribeMCL clustering results in a high average DCS (0.74).
(1.14 MB TIF)

Table S1 Marginal dependence between various orthology/homology detection methods.
(0.05 MB DOC)

Table S2 The performance of Reciprocal Best Hit (RBH) depends on the definition of ‘best-hit’.
(0.03 MB DOC)

Table S3 RSD performance varies according to divergence cutoff.
(0.03 MB DOC)
