# Supplementary figures and images for: Assessing Performance of Orthology Detection Strategies Applied to Eukaryotic Genomes
Source: PLoS One. 2007 Apr 18;2(4):e383. doi: 10.1371/journal.pone.0000383 (PMC1849888; doi:10.1371/journal.pone.0000383)

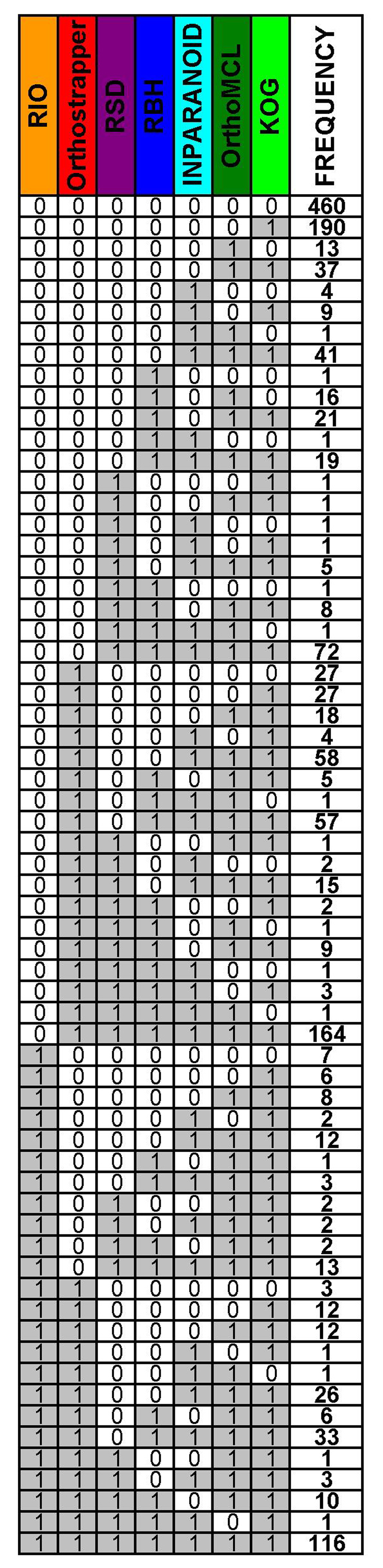

Supplement: Figure S1 — Frequency table from one sampling replicate. Last column lists the number of protein pairs observed for each orthology prediction pattern. Note that 50% (64) of the 2ˆ7 = 128 possible patterns are not observed in this replicate (the rows are not shown). (4.01 MB TIF) [file pone.0000383.s001.tif]

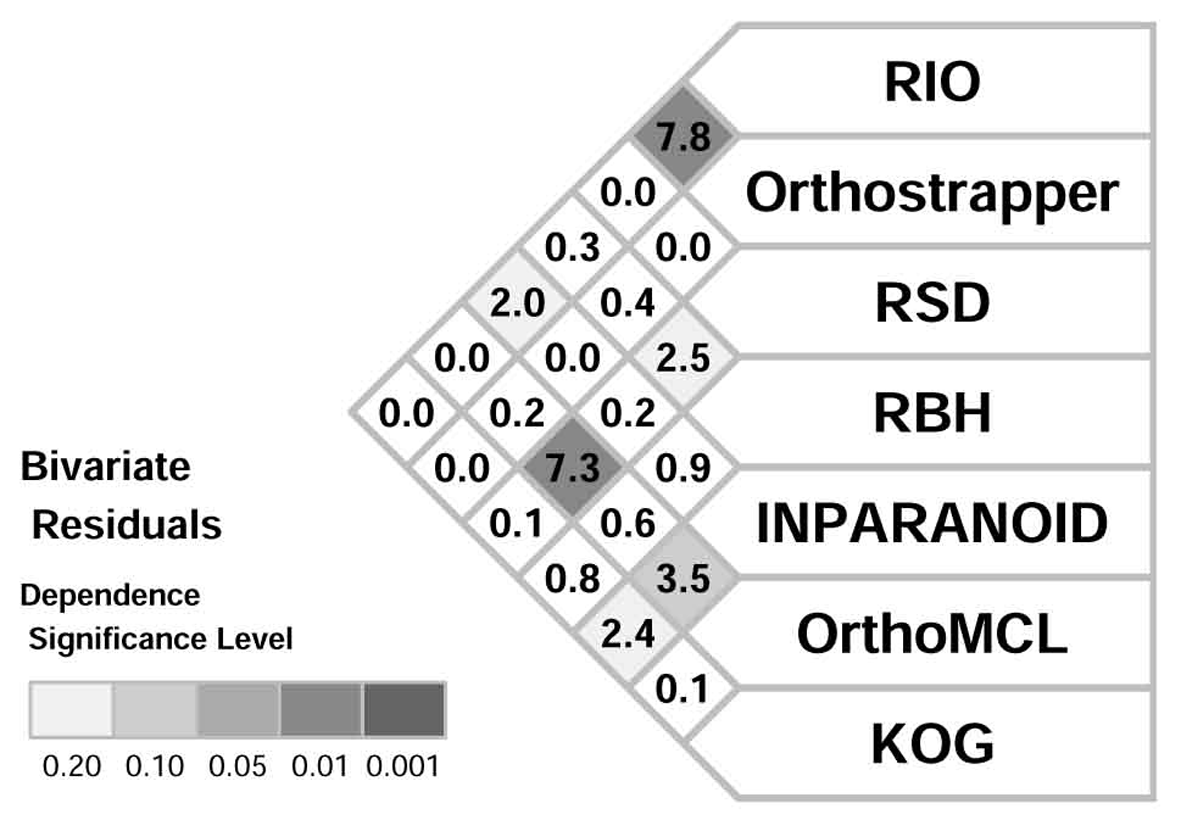

Supplement: Figure S2 — Bivariate residual statistics (BVR) calculated based on the CFactor 2LC latent class model. Note that most conditional dependencies decrease significantly in comparison with BVR statistics based on the 2LC model (Figure 3), indicating that they are effectively modeled by the extra latent variable added in the CFactor 2LC model. (0.42 MB TIF) [file pone.0000383.s002.tif]

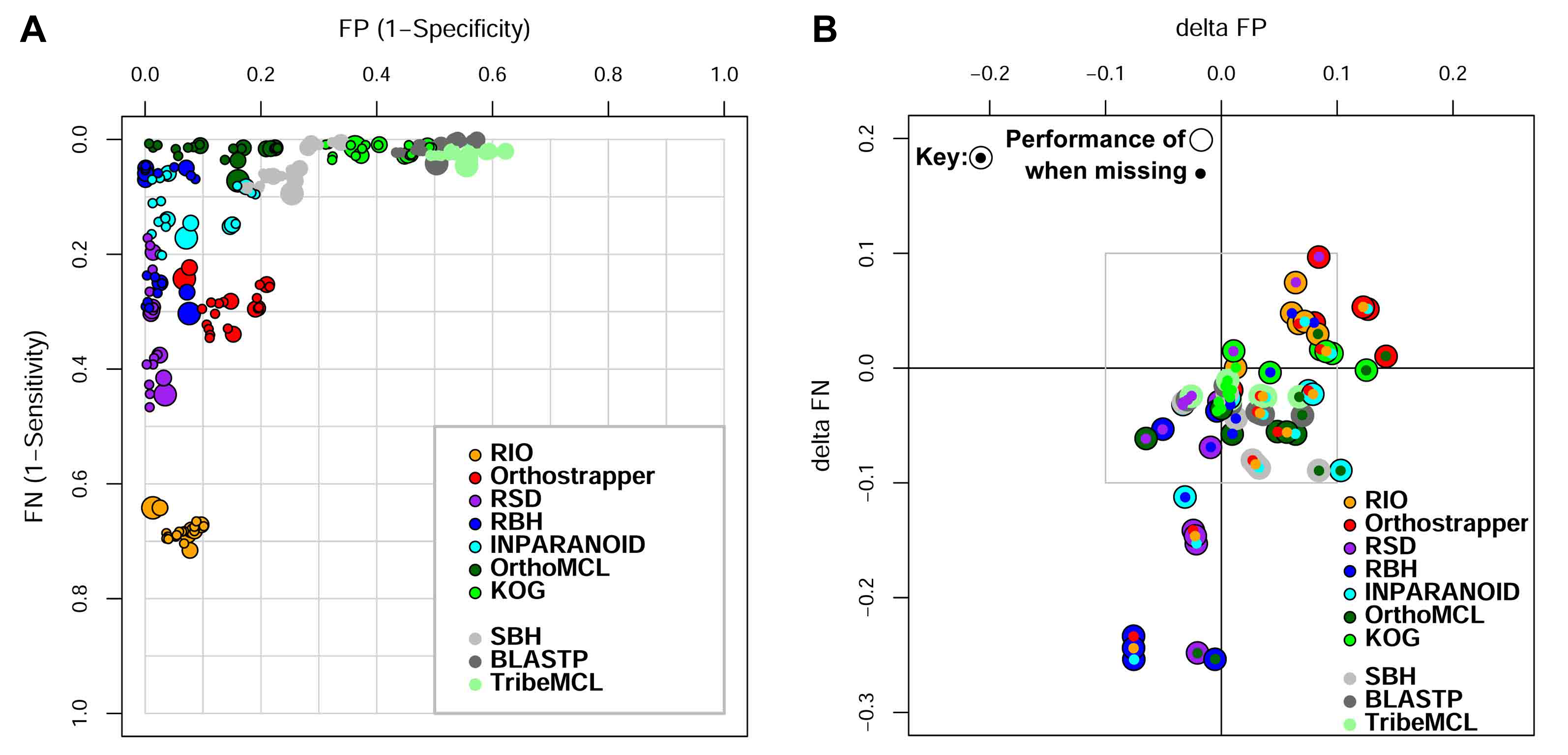

Supplement: Figure S3 — Jackknife analysis of orthology detection performance based on LCA. Panel A: Removal of any one or two orthology detection methods from the LCA benchmarking framework (medium- and small-sized circles, respectively) has relatively little impact on performance. Large circles indicate performance assessment when all methods are included (see Figure 3). Note that the overall trend of orthology detection performance is maintained in jackknife analysis. Panel B: Data extracted from panel A to indicate specific effects of (one-) method removal. Data points indicate changes in the estimation of FP & FN error rates for each method (outer circle), with respect to the original benchmarking result, following removal of other methods (inner circle). Most changes observed on these error rates are ≤0.1 (indicating the relative robustness in estimation), but systematic changes of some methods indicate possible errors: RBH's lower FN rates when some methods are removed suggest possible underestimation of sensitivity; phylogeny-based methods RIO and Orthostrapper are concentrated in the upper right quadrant, suggesting possible overestimation of performance. (2.72 MB TIF) [file pone.0000383.s003.tif]

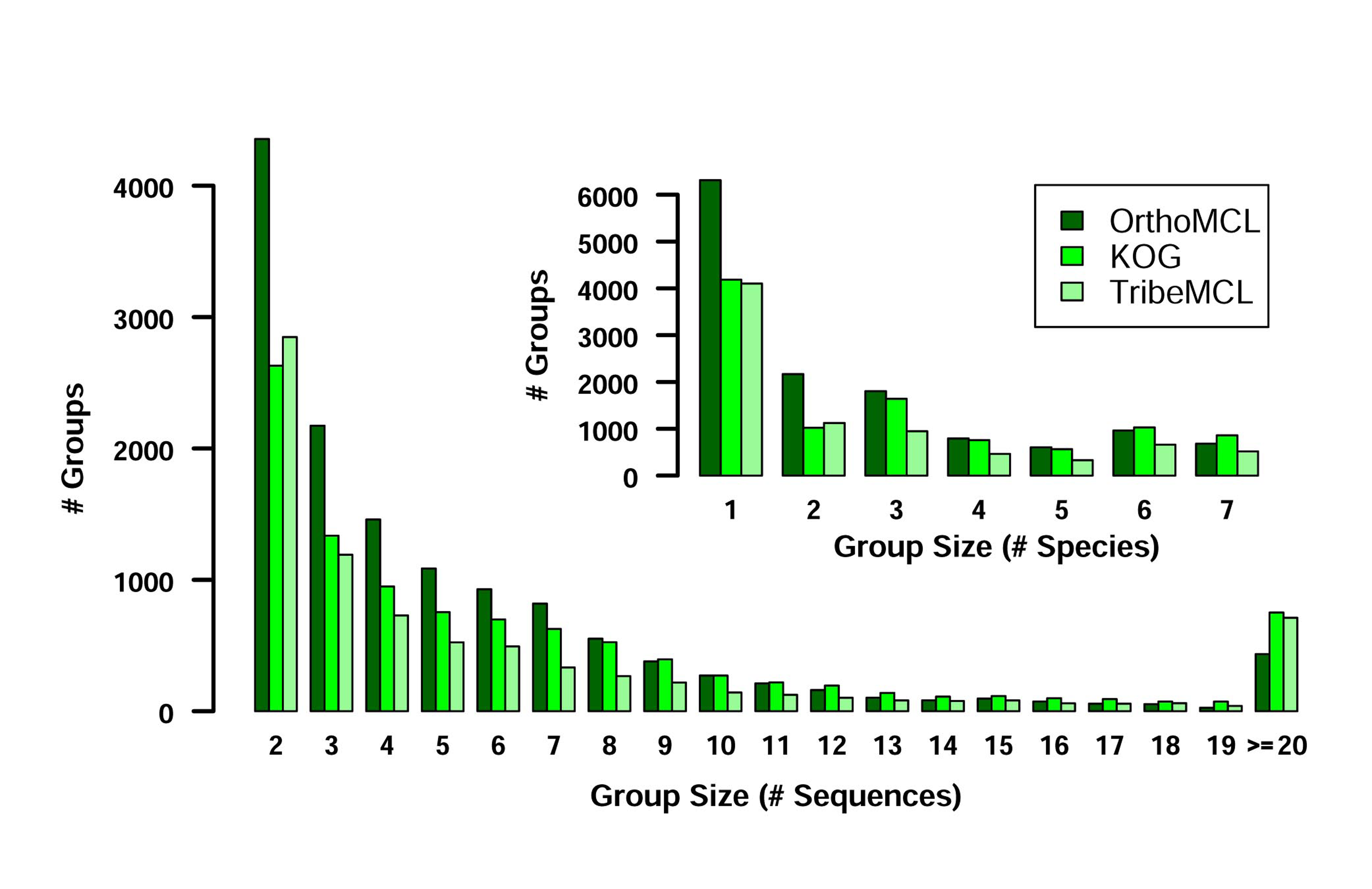

Supplement: Figure S4 — Size distribution of KOG, OrthoMCL and TribeMCL groups (with respect to # sequences and # species). When compared with KOG and TribeMCL, the majority of extra groups identified by OrthoMCL contain≤4 sequences, from 1–2 species. (2.05 MB TIF) [file pone.0000383.s004.tif]

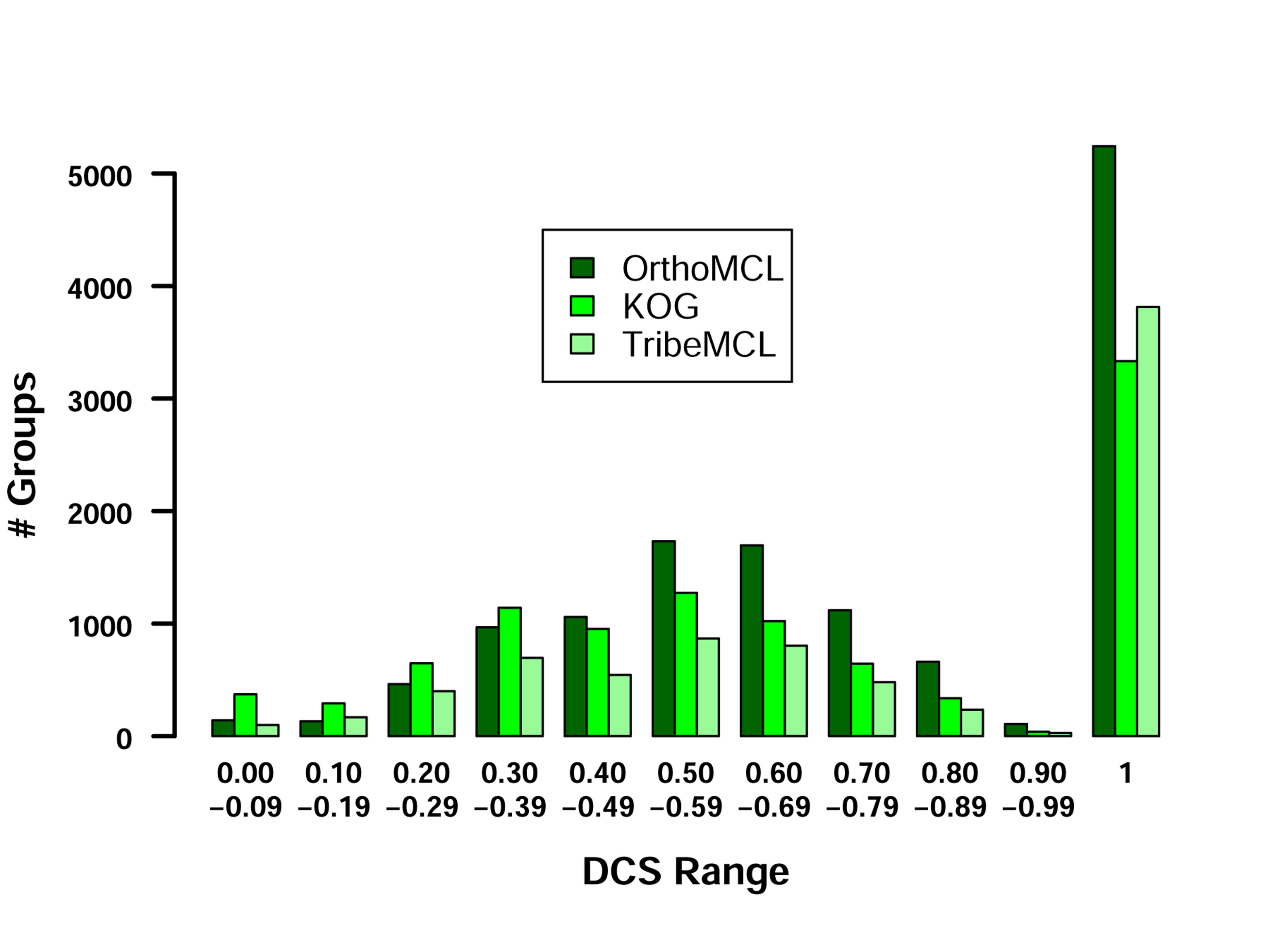

Supplement: Figure S5 — Domain structure consistency of OrthoMCL, KOG and TribeMCL groups. DCS (Domain Content Similarity) is defined as a Jaccard coefficient: the fraction of MKDOM2 domains found in either of two sequences that are present in both; DCS for a group of sequences is calculated as the average of all pairwise comparisons of sequences. (Note that the effects of domain repeat, order and length are not considered under this simple definition. In addition, due to the difficulties of accurately predicting gene models, e.g. start codon positions and intron/exon structures, orphan domains appearing only once in the entire dataset are excluded.) Groups from all three methods display a similar distribution in DCS value, with OrthoMCL exhibiting better consistency than KOG (average DCS is 0.73 for OrthoMCL vs 0.65 for KOG). The stringent BLAST E-value cutoff (10ˆ-10) used in TribeMCL clustering results in a high average DCS (0.74). (1.14 MB TIF) [file pone.0000383.s005.tif]
