# Supplementary material for: Assessing Performance of Orthology Detection Strategies Applied to Eukaryotic Genomes
Source: PLoS One. 2007 Apr 18;2(4):e383. doi: 10.1371/journal.pone.0000383 (PMC1849888; doi:10.1371/journal.pone.0000383)
Supplement: Table S1 — Marginal dependence between various orthology/homology detection methods. (0.05 MB DOC) [file pone.0000383.s006.doc]

**Table S1.** **Marginal dependence between various orthology/homology detection methods**

| **MI^a^ CCs^b^** | **RIO** | **Ortho-**  **strapper** | **RSD** | **RBH** | **Inpara-**  **noid** | **Ortho-**  **MCL** | **KOG** | **SBH** | **BLASTP** | **TribeMCL** |
| --- | --- | --- | --- | --- | --- | --- | --- | --- | --- | --- |
|
| **RIO** |  | 0.07 | 0.03 | 0.04 | 0.05 | 0.07 | 0.06 | 0.05 | 0.03 | 0.02 |
| **Orthostrapper** | 0.38 |  | 0.10 | 0.13 | 0.16 | 0.16 | 0.13 | 0.13 | 0.07 | 0.05 |
| **RSD** | 0.24 | 0.44 |  | **0.28** | 0.20 | 0.19 | 0.13 | 0.20 | 0.12 | 0.07 |
| **RBH** | 0.29 | 0.50 | **0.72** |  | 0.24 | **0.31** | 0.16 | **0.30** | 0.16 | 0.11 |
| **Inparanoid** | 0.31 | 0.56 | 0.61 | 0.67 |  | **0.35** | 0.22 | **0.26** | 0.17 | 0.14 |
| **OrthoMCL** | 0.34 | 0.55 | 0.57 | **0.71** | **0.77** |  | **0.26** | **0.31** | 0.21 | 0.18 |
| **KOG** | 0.30 | 0.48 | 0.44 | 0.51 | 0.60 | **0.67** |  | **0.25** | 0.18 | 0.17 |
| **SBH** | 0.30 | 0.49 | 0.55 | **0.68** | **0.67** | **0.74** | **0.67** |  | **0.31** | 0.20 |
| **BLASTP** | 0.22 | 0.34 | 0.40 | 0.48 | 0.52 | 0.60 | 0.61 | **0.71** |  | **0.34** |
| **TribeMCL** | 0.19 | 0.30 | 0.34 | 0.42 | 0.48 | 0.55 | 0.58 | 0.60 | **0.82** |  |

^a^MI, mutual information. The mutual information between variables *A* and *B* (in this study, *A* and *B* represent two methods’ prediction results) is calculated as $MI(A,B)=H(A)+H(B)-H(A,B)$ where $H(A)=-\sum p(a)\ln p(a)$ and $H(A,B)=-\sum\sum p(a,b)\ln p(a,b)$ [$p(a)$ and $p(a,b)$ are marginal and joint probability distributions, respectively]. The ten highest values are underlined.

^b^CCs, Pearson correlation coefficients. The ten highest values are underlined.
