# Supplementary material for: Assessing Performance of Orthology Detection Strategies Applied to Eukaryotic Genomes
Source: PLoS One. 2007 Apr 18;2(4):e383. doi: 10.1371/journal.pone.0000383 (PMC1849888; doi:10.1371/journal.pone.0000383)
Supplement: Table S2 — The performance of Reciprocal Best Hit (RBH) depends on the definition of ‘best-hit’. (0.03 MB DOC) [file pone.0000383.s007.doc]

**Table S2. The performance of Reciprocal Best Hit (RBH) depends on the definition of ‘best-hit’**

| **RBH Rules** | **Best E-value (<1e-99=0)** | **Best E-value (<1e-180=0)** | **Best Score** | **Best BitScore** | **Top One** |
| --- | --- | --- | --- | --- | --- |
| **FP** | 0.07 | 0.05 | 0.04 | 0.04 | 0.04 |
| **FN** | 0.30 | 0.36 | 0.38 | 0.38 | 0.41 |

Different rules for defining ‘best-hit’ result in different levels of ties (i.e. hits with the same value), therefore having different levels of stringency. The default rule analyzed in LCA analysis is “Best E-value” based on the BLAST result file downloaded from the KOG database (which has an underflow to zero when E-value is less than 1e-99); the ‘1e-180’ one is based on a separate BLAST run using NCBI-BLAST 2.2.13. The ‘Top One’ rule arbitrarily picks only the top BLAST hit, even when the second hit has an identical E-value or score (sometimes even the identical sequence).
