# Supplementary material for: Assessing Performance of Orthology Detection Strategies Applied to Eukaryotic Genomes
Source: PLoS One. 2007 Apr 18;2(4):e383. doi: 10.1371/journal.pone.0000383 (PMC1849888; doi:10.1371/journal.pone.0000383)
Supplement: Table S3 — RSD performance varies according to divergence cutoff. (0.03 MB DOC) [file pone.0000383.s008.doc]

**Table S3. RSD performance varies according to divergence cutoff**

| **RSD Divergence Cutoff** | **0** | **0.2** | **0.5** | **0.8 (default)** | **1** |
| --- | --- | --- | --- | --- | --- |
| **FP** | 0.01 | 0.04 | 0.04 | 0.04 | 0.04 |
| **FN** | 0.92 | 0.44 | 0.44 | 0.44 | 0.44 |

RSD uses a divergence cutoff to filter BLAST hits for distance calculations. For two sequences, the divergence is defined as the fraction of a pairwise alignment’s total length that is not alignable. Increasing stringency from 1 to 0 improves specificity, but the intermediate thresholds exhibit virtually identical performance.
